# Risk Factors Associated With the Development of Calciphylaxis in Patients With Chronic Kidney Disease: A Systematic Review

**DOI:** 10.7759/cureus.75314

**Published:** 2024-12-08

**Authors:** Cyprian O Ogah, Huda Mohammed, Ingie M Gabra, Nouran Halawa, Saira Naeem, Safeera Khan

**Affiliations:** 1 Internal Medicine, Valley Baptist Medical Center Harlingen, Harlingen, USA; 2 Colorectal Surgery, Luton and Dunstable University Hospital, Luton, GBR; 3 Anesthesiology, John Muir Health, Walnut Creek, USA; 4 Internal Medicine, Ain Shams University, Cairo, EGY; 5 Medicine, Faisalabad Medical University, Faisalabad, PAK; 6 Internal Medicine, California Institute of Behavioral Neurosciences and Psychology, Fairfield, USA

**Keywords:** calcific uremic arteriolopathy (cua), calciphylaxis, chronic kidney disease, chronic renal failure, dialysis, dm, end stage renal disease, pth, risk factors, vka

## Abstract

Calciphylaxis is a rare but potentially life-threatening disease that is not yet completely understood. It occurs mainly in patients with chronic kidney disease termed calcific uremic arteriolopathy (CUA) but also affects patients with normal renal function. Although this disease's pathogenesis is unclear, it is associated with the dysregulation of calcium and phosphate and subsequent calcification of peripheral arterioles. Calciphylaxis has up to 80% mortality, even with multidisciplinary and multimodal treatment modalities.

The available literature identified some relevant risk factors associated with the development of calciphylaxis, but the authors differ significantly in risk profiling. Moreover, most papers on calciphylaxis are observational studies, namely case series, case reports, cohort, and cross-sectional studies. Although recently available articles mentioned some risk factors, the studies mainly focused on diagnosis, treatment, and prognosis with limited emphasis on structured risk profiling. In addition, experimental and systemic review studies on risk factors associated with calciphylaxis are lacking. Thus, this systematic review's primary focus is to determine risk factors associated with developing calciphylaxis in patients with chronic kidney disease.

We searched electronic databases from 2018 to 2024 for articles that contained relevant risk factors linked with the development of calciphylaxis using the keywords calciphylaxis, chronic kidney disease, and risk factors. We identified 486 articles, removed duplicate papers from selected articles, applied inclusion and exclusion criteria, and conducted a quality assessment test. Two independent authors performed data extraction manually, and we compared the results for consistency and accuracy.

Twenty-two articles met the eligibility criteria, but only 16 articles passed the quality assessment appraisal and were included in the systematic review. We identified 1,739 patients: 664 (38.2%) and 1075 (61.81%) were males and females, respectively. A total of 1373 (78.95%) were on dialysis, and 926 (53.25%) were diabetics. Caucasians and patients with obesity were 695 (37.90%) and 613 (35.25%), respectively. At the time of diagnosis, 599 (34.45%), 311 (17.90%), and 278 (15.99%) were on vitamin K antagonists (VKA), vitamin D, and calcium phosphate binders. The number of patients with elevated parathyroid hormone was 196 (11.27%).

Our study found no experimental or systematic reviews that primarily focused on risk factors associated with the development of calciphylaxis. Our research indicated that dialysis is the most frequent risk factor linked to the development of calciphylaxis. Other risk factors include being caucasian, female gender, obese, diabetic mellitus (DM), having elevated parathyroid hormone, and use of VKA (warfarin), vitamin D, and calcium phosphate binders. These findings are consistent with the evidence seen in most of the articles we reviewed. However, the papers we studied are mainly observational mono-centered research articles, with the majority having a small sample size. Thus, we recommend a multicenter, large-scale experimental study to assess risk factors and profiling for the development of calciphylaxis in patients with chronic kidney disease.

## Introduction and background

Calciphylaxis, which is commonly known as calcific uremic arteriolopathy (CUA), is a rare and potentially life-threatening condition that remains incompletely understood [1]. It occurs mainly in patients with chronic kidney diseases but can also affect patients with normal kidney function [2,3]. The one-year survival rate varies between 45% and 55% [4], with annual mortality estimated at 45%-80%. The high mortality rate of calciphylaxis is primarily attributed to complications of sepsis and wound infections [2,5]. Calciphylaxis has variable clinical presentation, the main factor contributing to delays in prompt diagnosis and timely treatment, resulting in poor clinical outcomes [5].

This pathology, later termed calciphylaxis, was first described by Bryant and White in 1898 in patients with chronic kidney disease [3]. However, it was not until 1961 that Professor Hans Selye and his colleagues coined calciphylaxis following a laboratory induction of generalized subcutaneous soft tissue calcification in rats [2]. Hans and his colleagues used challenging agents such as high parathyroid hormone, high dose Vitamin D, and high dose phosphate diet on the experimental animals (rats). During the experiment, they found that calciphylaxis in these animals did not involve blood vessels; also, they observed a resolution of the systemic calcification upon discontinuing the offending agents [2]. Thus, they considered calciphylaxis a hypersensitive reaction to the offending agents. This finding in experimental animals differs from humans in that in humans, calcification is an accelerated vascular and extraosseous calcification of small arteries and arterioles, resulting in the osteogenic transformation of vascular smooth walls [2].

Many authors have identified several risk factors associated with the development of calciphylaxis; however, accurate diagnosis remains a challenge, and this contributes to an overall poor treatment prognosis [6]. Although calciphylaxis was previously considered a rare disease, the incidence is rising and has drawn attention worldwide in the last decade [7]. With the unclear pathogenesis of calciphylaxis, understanding the associated risk factors may help define the exact etiology [7]. Also, with the increasing prevalence, improving clinical understanding of calciphylaxis and different diagnostic protocols that can improve screening and treatment outcomes are necessary [8]. Interestingly, it is worrisome that in addition to difficulty in accurate preventive and diagnostic modalities, treatment protocols are not yet standardized, further complicating the challenges in achieving optimal treatment outcomes [9].

Although clinicians still face difficulty in diagnosing calciphylaxis, there is increased accuracy due to a clearer understanding of risk factors and more reliable histologic diagnostic protocols in recent years [10]. Nevertheless, diagnosis still requires a high index of suspicion and clinical vigilance because calciphylaxis mimics many other skin lesions, such as peripheral vascular disease, skin infections, and ulcers [2,10]. Skin biopsy is the standard diagnostic procedure, but biopsy results must be strengthened with clinical presentation and laboratory abnormalities involving calcium, parathyroid hormone (PTH), phosphate, and vitamin D [10]. Although abnormal histopathologic findings from skin biopsies seen in patients with chronic kidney disease usually support the validity of clinically suspected calciphylaxis, these findings are unreliable as similar findings can also be seen in non-CUA skin biopsies [11].

Despite recent advances in understanding calciphylaxis's diagnosis and risk factor stratification, the exact pathogenesis remains complex and challenging [12]. Most authors have identified the presence of abnormalities in calcium and phosphate metabolism resulting in calcium deposition in the intima and media of arteries and arterioles as fundamental to the pathogenesis of calciphylaxis. Also, they noted that patients of the female gender, Caucasian race, hyperparathyroidism, use of vitamin K antagonists (VKA), and those patients on dialysis [1,2,10,12] are at increased risk for developing calciphylaxis.

Worse still, even though calciphylaxis has high mortality and morbidity, there is no approved standard diagnostic tool or licensed treatment modality at present; also, no singular treatment protocol guarantees treatment success. Thus, a multifaceted treatment strategy is recommended [13]. Again, CUA applies to patients with chronic kidney disease only; however, calciphylaxis can also affect patients with normal renal function. Thus, using the term CUA to refer to all forms of calciphylaxis appears ambiguous. This concept contributed further to the inherent difficulties in understanding and managing calciphylaxis [14]. Moreover, the unreliable and nonspecific histological diagnostic findings from skin biopsies from patients with calciphylaxis also add to the growing complexity of understanding and managing calciphylaxis. This is true because patients with skin lesions from different pathologies, such as peripheral vascular disease, may have similar histologic biopsy results to calciphylaxis.

Furthermore, due to the rarity of calciphylaxis and the limited understanding of the disease, the rate of diagnosing calciphylaxis using skin biopsy as the gold-standard diagnostic modality is low [15]. This is because findings on skin biopsy are not specific to calciphylaxis since several other skin lesions have histopathological results similar to calciphylaxis. This disease usually presents as non-healing ulcerative plaques and necrosis, mainly involving the extremities. This is due to pathophysiologic changes that stimulate endovascular fibrosis, vascular thrombosis, tissue ischemia, infarction, calcification, and infection, primarily due to inadequate blood supply to extremities compared to other areas of the body [16].

## Review

Methodology

Search Strategy

We searched medical databases, including PubMed, Pub Mesh, PubMed Advance, EBSCO library, Cochrane Library, PMC, and Science Direct, for relevant articles that contained risk factors related to calciphylaxis. In PubMed Advance, we used (calciphylaxis [Title/Abstract]) AND (dialysis [Text Word])) AND ("chronic kidney disease" [Text Word]) strategy. In Pub Mesh, ("Calciphylaxis/etiology" [Majr]OR "Calciphylaxis/mortality" [Majr] OR "Calciphylaxis/prevention and control" [Majr]) AND ("Renal Dialysis/adverse effects" [Majr] OR "Renal Dialysis/mortality" [Majr]) AND ("Renal Insufficiency, Chronic/etiology" [Majr] OR "Renal Insufficiency, Chronic/mortality" [Majr] OR "Renal Insufficiency, Chronic/prevention, and control" [Majr]) were used in search strategy. Additionally, we employed Boolean operators (AND and OR) to search for articles from the rest of the databases.

Inclusion and Exclusion Criteria

We selected papers published only in English within the last six years; full text is available. The selected documents must involve human subjects between 19 and 70 years old. All the papers must contain only male and female genders and not be limited to geographic areas. We excluded articles authored by one person, published before 2018, and containing less than 80% of the relevant risk factors under study.

Selection Process

**Figure 1:**
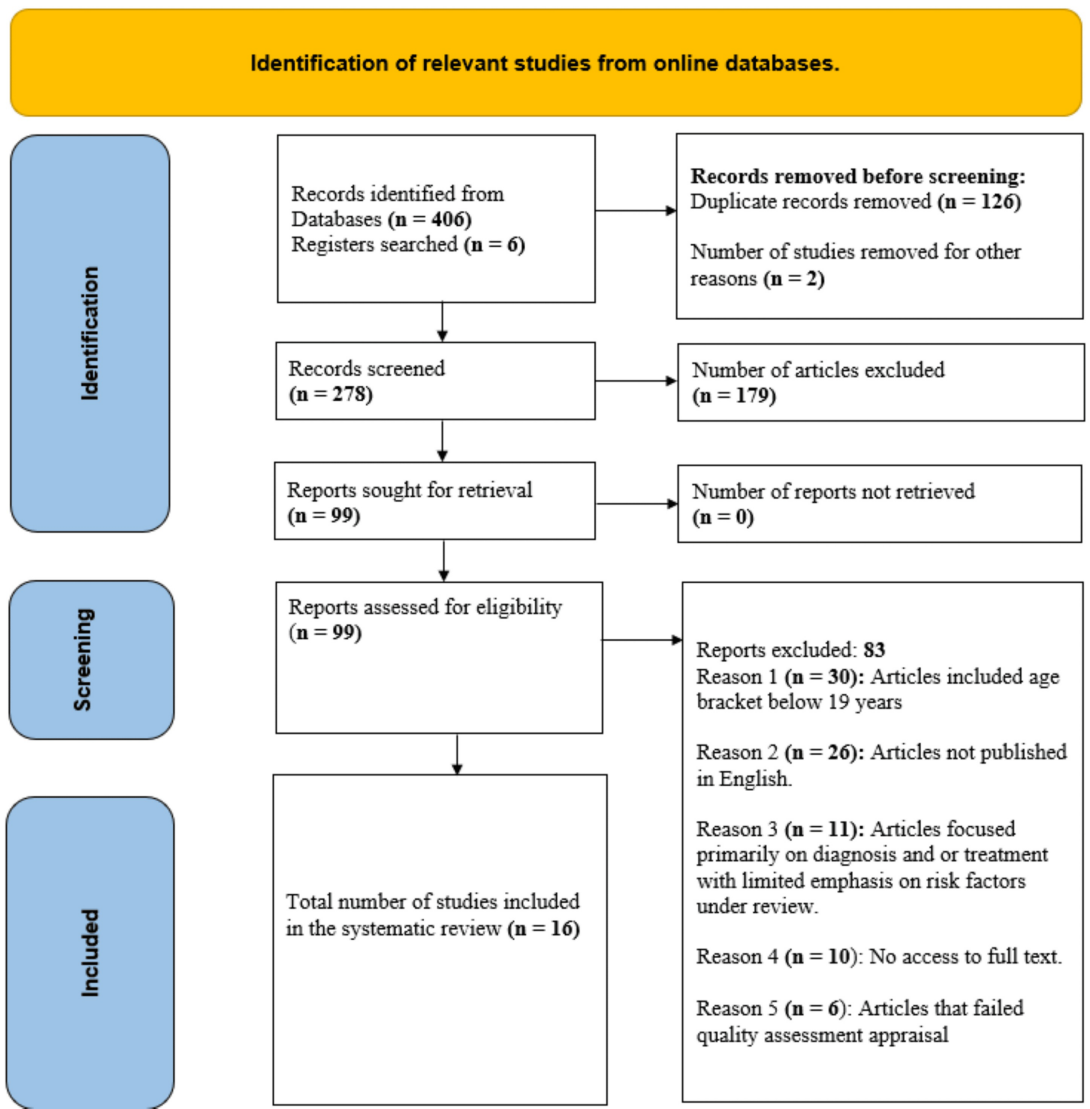
A PRISMA flow chart for studies selection. PRISMA: Preferred Reporting Items for Systematic Review and Meta-Analysis

Before removing duplicate articles, we transferred identified papers during online data search to EndNote (Clarivate, Philadelphia, USA). Articles that met inclusion and exclusion criteria were assigned to Microsoft Word (Microsoft® Corp., Redmond, WA, USA) by the authors. The titles, abstract, and conclusion of each selected article were assessed by Cyprian Ogah Omeh (the primary author) using already agreed eligibility, inclusion, and exclusion criteria. We excluded papers that did not contain relevant information of interest or had a bias not fully addressed by the authors after reading the full text. We identified 406 articles, but only 22 studies met eligibility, inclusion, and exclusion criteria, as shown in the Preferred Reporting Items for Systematic Review and Meta-Analysis (PRISMA) flow diagram (Figure 1) [17]. Finally, we reviewed 16 articles that passed the quality assessment appraisal. Following an online database search, we presented the selected studies in the PRISMA chart [17], as shown in Figure 1.

Quality Assessment Check

First, we applied the Strengthening the Reporting of Observational Studies in Epidemiology (STROBE) tool to assess the quality of the articles we selected for review [18]. Then, we used Joanna Briggs Institute (JBI) to conduct quality appraisals for case series, cohorts, and case reports [19]. Likewise, we assessed systematic reviews and meta-analyzed articles using Assessing the Methodological Quality of Systematic Reviews (AMSTAR) [20]. The result yielded only 16 articles that were used in our systematic review.

Data Extraction

After selecting 16 articles for review, we read the full text of each paper, extracted relevant information, and transferred the data to an Excel spreadsheet for further analysis. Tables 1-2 show the summary characteristics of the 16 articles in the systematic review.

**Table 1:** The table represents the characteristic findings from each of the articles reviewed. ESRD: end-stage renal disease; PTH: parathyroid hormone

S. no.	Name of the author	Year of the publication	Type study	Sample size	Purpose of the study	The outcome of the study	Limitations and recommendations
1	Lajoie et al. [1]	2023	Case series	12	Multimodal treatment approach to calciphylaxis in patients with ESRD	75% of patients responded to treatment with 45% mortality per year	The study is limited by a small sample size, which may affect the statistical power
2	Ruderman et al. [2]	2021	Case series	47	Clinical features and treatment outcomes in patients with calciphylaxis	50% mortality, even with a multimodal treatment protocol	Most of the centers that participated in the research failed to report clinical data
3	Turek et al. [3]	2021	Case report	1	Multifactorial pathogenesis of calciphylaxis	Early diagnosis and prompt treatment improve outcome	A small sample size, which is prone to limited power
4	Gaisne et al. [4]	2020	Case report	89	Datamine epidemiological characteristics, risk factors, treatment, and survival	Weight loss and parathyroidectomy worsen calciphylaxis	The method of data collection is cross-sectional
5	Toussaint et al. [5]	2024	Case series	333	Assessment of associated risk factors and treatment outcomes of calciphylaxis	Increased incidence of calciphylaxis among the Māori population compared to the Caucasian race	The validity of the finding is questionable as Māori population has never been involved in similar studies
6	Wen et al. [6]	2023	Case series	111	Assess pain intensity about clinical improvement and outcomes	Improvement in pain reduces the risk of amputation	Difficulty in accurately measuring pain threshold among patients
7	Liu et al. [7]	2021	Case-control	20	Describe risk factors for the development of calciphylaxis	PTH, warfarin, and female gender are not significant risk factors	A small sample size limits the power
8	Liu et al. [8]	2022	Cross-sectional	48	Prevalence and clinical characteristics of calciphylaxis	Prevalence is 1.24% in the population studied	Findings could be affected by a limited understanding of calciphylaxis in the Chinese population
9	Udomkarnjananun et al. [9]	2018	Systemic review/meta-analysis	856	Determine treatment modalities	Available treatment options offer little to no benefit	Limited sample size may affect the accuracy of the finding

**Table 2:** The table represents the characteristic findings from each of the articles reviewed (continued). CKD: chronic kidney disease; CUA: calcific uremic arteriolopathy; VKA: vitamin K antagonist; DOAC: direct oral anticoagulant; PTH: parathyroid hormone

S. no.	Name of the author	Year of the publication	Type study	Sample size	Purpose of the study	The outcome of the study	Limitations and recommendations
9	Udomkarnjananun et al. [9]	2018	Systemic review/meta-analysis	856	Determine treatment modalities	Treatment options offer little to no benefit	Limited sample size may affect the accuracy of the finding
10	Sánchez-Pujol et al. [10]	2021	Case series	16	Determine risk factors and diagnostic findings	75% of patients died from sepsis	Bias is likely due to the study type and limited sample size.
11	Røndbjerg et al. [11]	2023	Cross-section	9	Describe the presence of small vessel calcification in non-lesional tissues in patients with different stages of CKD.	CUA typically involves areas of the body with abundant cutaneous and subcutaneous tissues	A small sample size which is prone to limited power. Screening modality (skin biopsy) is unreliable as many other skin lesions can mimic CUA.
12	Panchal et al. [12]	2020	Retrospective review	30	Datamine the concomitant risk factors, treatment effectiveness, and prognosis.	No significant survival benefit with use of sodium thiosulphate (STS)	Absence of control treatment group may cause bias in the findings noted.
13	Chinnadurai et al. [13]	2014	Retrospective chart review	89	Assessment of treatment outcomes of calciphylaxis patients with end-state renal disease (ESRD)	No significant treatment benefit was observed using any specific treatment modality	A multifaceted treatment modality is recommended.
14	Yousuf et al. [14]	2024	Literature review	39	Assess clinical characteristics and treatment modalities	Amino-derived mesenchymal stem cell treatment modality appears promising	The study is prone to bias due to poor treatment follow-up. Larger sample size is recommended
15	Russ et al. [15]	2019	Retrospective study	15	Determine the pathogenesis of calciphylaxis	VKA is a risk factor. DOAC is more beneficial than VKAs as anticoagulant	The power is limited by small sample size and non-blinding of observers. Large sample size is recommended
16	Omer et al. [16]	2021	Case series	24	Assess effective treatment modality and diagnosis of calciphylaxis	Patients with low PTH and calcium phosphate binders have better treatment outcome	Early referral to nephrology and multimodal treatment approaches are recommended.

We extracted data on relevant risk profiles/factors from the 16 articles we reviewed and analyzed the most frequently occurring risk factors in patients with calciphylaxis and chronic kidney disease. Tables 3-4 summarize patients' risk profiles and epidemiological characteristics from the reviewed articles.

**Table 3:** The table represents the clinical characteristics of the study populations described in the reviewed articles. DM: diabetes mellitus; VKA: vitamin K antagonist; PTH: parathyroid hormone; Ca++: calcium

S. no.	Name of the author	Year of the publication	Type study	Sample size (N)	Mean age (yrs)	Caucasia (N)	Male (N)	Female (N)	Obesity (N)	DM (N)	Dialysis (N)	VKA use (N)	High PTH (N)	Vitamin D use (N)	Ca++ binders use (N)
1	Lajoie et al. [1]	2023	Case series	12	64	8	4	8	7	11	9	9	12	0	10
2	Ruderman et al. [2]	2021	Case series	47	55	43	23	24	-	35	29	16	-	19	20
3	Turek et al. [3]	2021	Case report	1	52	1	0	1	1	1	1	1	1	1	1
4	Gaisne et al. [4]	2020	Case report	89	52	89	32	57	89	1	70	64	58	72	47
5	Toussaint et al. [5]	2024	Case series	333	63	301	155	178	196	219	319	-	-	-	-
6	Wen et al. [6]	2023	Case series	111	58	76	44	67	-	68	88	66	7	84	62
7	Liu et al. [7]	2021	Case control	20	64	-	16	4	10	9	20	3	13	5	13
8	Liu et al. [8]	2022	Cross-sectional	48	54	48	33	15	21	48	48	4	35	-	-
9	Udomkarnjananun et al. [9]	2018	Systemic review & meta-analysis	856	56	-	257	599	239	419	651	342	-	46	40

**Table 4:** The table represents the clinical characteristics of the study populations described in the reviewed articles (continued). DM: diabetes mellitus; VKA: vitamin K antagonist; PTH: parathyroid hormone; Ca++: calcium

S. no.	Name of the author	Year of the publication	Type study	Sample size (N)	Mean age (yrs)	Caucasian (N)	Male (N)	Female (N)	Obesity (N)	DM (N)	Dialysis (N)	VKA use (N)	High PTH (N)	Vitamin D use (N)	Ca++ binders use (N)
10	Sánchez-Pujol et al. [10]	2021	Case series	16	68	-	6	10	3	4	6	10	3	-	12
11	Røndbjerg et al. [11]	2023	Cross-sectional	9	69	-	6	3	-	4	9	1	-	-	2
12	Panchal et al. [12]	2020	Retrospective review	30	66	27	8	22	17	20	23	10	29	16	16
13	Chinnadurai et al. [13]	2014	Retrospective chart review	89	59	85	35	54	-	48	78	37	-	60	14
14	Yousuf et al. [14]	2024	Literature review	39	50	-	33	6	18	19	24	23	23	12	22
15	Russ et al. [15]	2019	Retrospective study	15	54	15	6	9	12	7	3	13	13	10	7
16	Omer et al. [16]	2021	Case series	24	56	2	6	18	-	13	22	-	22	-	10

**Figure 2:**
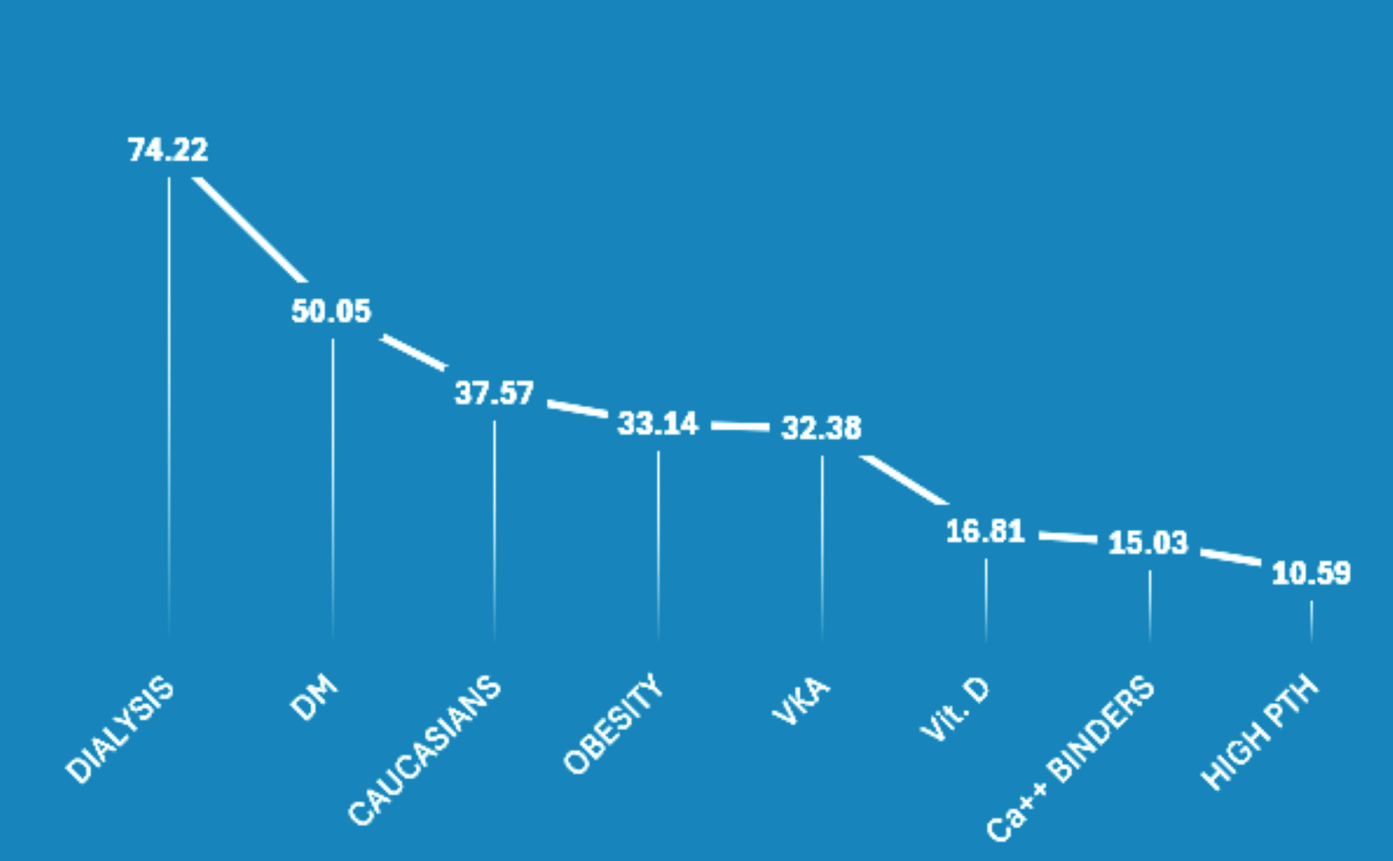
A graphic representation of the most common risk factors associated with the development of calciphylaxis in patients with chronic kidney disease. DM: diabetes mellitus; VKA: vitamin K antagonist; PTH: parathyroid hormone; Ca++: calcium NB: The numbers on the graph are in percentages.

From each reviewed article, we studied the most frequently occurring risk factors associated with the development of calciphylaxis in patients with chronic kidney disease. The findings are presented in Figure 2.

**Figure 3:**
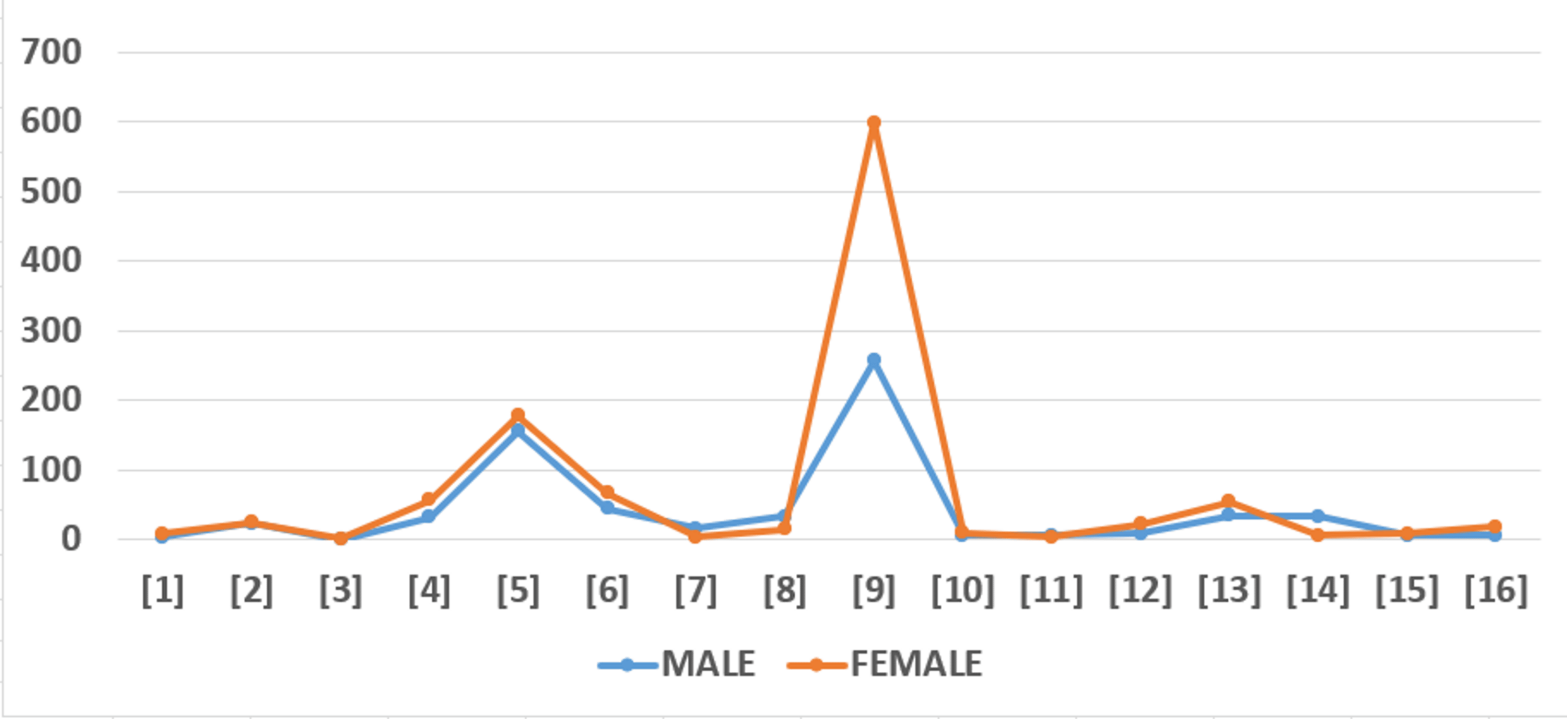
The figure represents the distribution of males and females in each of the reviewed articles. The numerical numbers inside each square bracket along the X-axis represent the articles studied.

We found an interesting pattern of male-to-female risk frequency associated with the development of calciphylaxis, as shown in Figure 3.

Discussions

Once calciphylaxis is developed, diagnosis and treatment are challenging, resulting in poor treatment outcomes and high mortality rates. These challenges underscore the need to prevent calciphylaxis from developing by establishing preventive modalities through identifying and actively managing risk factors [2]. Yes, several articles have identified dialysis, Caucasians, female gender, obesity, DM, hypertriglyceridemia, hypoalbuminemia, atherosclerosis, elevated PTH, and use of VKA and calcium-based phosphate binders as risk factors for the development of calciphylaxis. Nevertheless, none of the studies fully characterized these risk factors. Besides, all the studies are observational, with most papers having a small population sample size. While systematic research will improve understanding of the risk profile associated with calciphylaxis, a multicenter experimental study involving a large sample size is necessary.

Relevant Risk Factors Studied

Gender: Female gender is a historically known risk factor associated with the development of calciphylaxis. The result from our review is consistent with this fact. For example, Toussaint et al.'s study [5] is consistent with the evidence that calciphylaxis affects females more than males. However, the authors noted that the difference in the number observed between males and females is narrow. Nevertheless, they argued that since the study is recent and has a large sample size, its clinical relevance needs to be considered.

Similarly, a case study by Liu et al. [7] in a Chinese health institution indicated female-male-predominance, but like Toussaint et al.'s, the difference is not statistically significant. Nonetheless, the authors cautioned that the non-statistical significance of the article may be due to a limited understanding and study on calciphylaxis in the Chinese population. On the contrary, a retrospective literature review by Yousuf et al. on treatment modalities published in PubMed indicated that males are more affected than females [14], contradicting evidence found in other studies. Nevertheless, the author cautioned that this evidence may be biased since it is a single-center observational study with a small sample size. Again, the researchers argued that in addition to sample size, insufficient data gathering and scarce patient medical histories might limit the accuracy of the documented evidence.

Likewise, a cross-sectional study by Røndbjerg et al. [11] aligns with Yousuf's finding that supports male-to-female predominance. Still, Røndbjerg's research is a cross-sectional study with an even smaller sample size, predisposing the survey to a more likely bias. Conflicting evidence notwithstanding, most authors found that females are more predisposed to developing calciphylaxis than males.

Race: Like gender, most authors indicated that Caucasians are more likely to develop calciphylaxis than other races [2,4,5]. However, some of the reviewed articles have contrary evidence. For example, a tertiary institution-based case series study by Omer et al. [16] revealed that African-American descent has more calciphylaxis than their Caucasian counterpart. However, the author said the study center serves predominantly African-American patients. Again, they cautioned that the study's small sample size may be a source of bias. Thus, they recommended a multicenter controlled clinical trial. Similar to Mohamed et al., a calciphylaxis study in the Australian and New Zealand Dialysis and Renal Transplant Center by Toussaint et al. [5] found that even though calciphylaxis affects Caucasians more than other races, Maori ethnic patients in Australia and New Zealand were found to have a higher risk of calciphylaxis in comparison to their European counterparts.

Interestingly, unlike the small sample-size study conducted by Mohamed et al., the article authored by Toussaint et al. [5] involved a larger population size and is also more recent, which may increase the validity of the documented evidence. Nevertheless, Toussaint et al. argued that the evidence should be interpreted cautiously since Maori patients have never been included in similar studies. In summary, because of some authors' mixed reporting on the risk of calciphylaxis among different races, experimental research would be appropriate to correctly determine which race is more predisposed to developing calciphylaxis.

Dialysis: This remains the most frequently observed risk factor associated with the development of calciphylaxis in patients with chronic kidney disease [2,5,9]. Of course, this is understandable, given the fact that most patients with chronic kidney disease are likely to be on dialysis at some point along the continuum of care. Similarly, Omer et al. [16] reported that about 62% of the patients studied were on dialysis at the time the diagnosis of calciphylaxis was made. More interestingly, Toussaint et al. [5], in their study involving a clinical registry in Australia, found that 96% of the patients received one form of dialysis or the other at the time of diagnosis. Likewise, Wen et al. [6], in a case series research, indicated that 79% of the population studied were on dialysis. Nevertheless, a note of caution in interpreting these findings rests on the fact that though their patients had chronic kidney disease, 25% of the patients had normal renal function at the time of diagnosis. Thus, the authors recommended an experimental study to validate these findings.

Contrary to the above evidence on dialysis, Sánchez-Pujol et al. [10] published in Science Direct showed that less than 50 of the studied population was on dialysis at the point of diagnosis. This evidence is inconsistent with the findings documented in most articles we reviewed. Similarly, Russ et al. [15], in a retrospective study published in PubMed, mirrors Sánchez-Pujol et al. because the number of patients on dialysis at the time of diagnosis was less than 50%. However, unlike Sánchez-Pujol et al., the lack of blinding to the observers and the use of an unstandardized sample collection methodology adopted during data collection could limit the clinical significance of this evidence.

DM: DM is one of the common risk factors associated with the development of calciphylaxis. Our analysis shows that over 50% of the patients reviewed had DM at diagnosis. This finding is consistent with the evidence in most articles we reviewed, especially in a case series study by Ruderman et al. [2], which indicated that 76% of the patients had DM when calciphylaxis was diagnosed.

On the contrary, in a matched case-control study conducted by Liu et al. [7] involving the Chinese population, there was no significant difference in the risk frequency of DM between patients in the case and control group. Nonetheless, the authors suggested that the small sample size and indigenous and local contributing factors like limited awareness and knowledge of calciphylaxis may account for this difference compared to evidence commonly seen in European countries. Therefore, they advised that larger multicenter or experimental studies are needed to assess further the relationship between DM and the development of calciphylaxis. Likewise, Røndbjerg et al. [11], a cross-sectional study, indicated that the percentage of patients with DM is less than 50%, and even though the study is recent, it is cross-sectional with a small sample size, which may affect the accuracy of evidence as documented. Thus, like most authors, they recommended an extensive multicentered experimental study to determine the link between DM and the development of calciphylaxis.

Obesity: Several of the articles we reviewed indicated that obesity is a significant risk factor associated with the development of calciphylaxis. More importantly, the systematic review and meta-analysis captured the clinical importance of obesity as a common risk factor linked with calciphylaxis [9]. Interestingly, Gaisne et al. [4] found that unintentional weight loss is associated with the development of calciphylaxis, as documented in their case-control study conducted in France in 2020. Surprisingly, in our systematic review, only 35.25% of the patients were obese. This record contradicted the evidence documented in most of the articles we reviewed. We can attribute our finding to several factors, namely, the use of the average body mass index of the studied population instead of the absolute numerical value for obesity in the studies reviewed. Also, documenting weight in percentages necessitated us to convert percentages to numerical values, as seen in an article by Ruderman et al. [2]. Similarly, some articles failed to document the number of patients with obesity [2,6,11,13,16], respectively. We believe these inconsistencies and conversions in some of the articles may affect the accuracy of our systematic review findings.

Hyperparathyroidism: Elevated PTH, like other risk factors described above, is known to be associated with the development of calciphylaxis. According to Liu et al. [7], 65% of patients had elevated PTH when the diagnosis of calciphylaxis was established. Similarly, a cross-sectional survey conducted by the same author in 2020 in China showed that 73% of the patients with calciphylaxis had elevated PTH. Also, evidence documented by Gaisne et al. [4], Sánchez-Pujol et al. [10], and Russ et al. [15] showed that more than 50% of the patients in their various studies had elevated PTH. Contrary to this evidence, our review indicated that only 12.45% of patients had elevated PTH when calciphylaxis was diagnosed. Nevertheless, our finding may be attributed to inaccurate representation or missing data on elevated PTH, as seen in some articles reviewed [2,9].

VKA (warfarin): Warfarin, used synonymously with VKA in the reviewed article, is another significant risk factor linked with the development of calciphylaxis. According to Sánchez-Pujol et al. [10], 62.5% of patients were on warfarin at the time of diagnosis of calciphylaxis. Similarly, Liu et al. [7] indicated that VKA use increases the risk of calciphylaxis. However, the author did not find any clinical significance in this evidence mainly because the frequency of warfarin use among Chinese patients is low compared to their European or North American counterparts. Moreover, this case-control study has a small sample size and a short study duration. Thus, Liu et al. [7] recommended a multicentered experimental study to evaluate the link between the use of warfarin and the development of calciphylaxis. In our pooled data in this systematic review, 34.45% of patients were on VKA at the time of diagnosis of calciphylaxis. This result contradicted the evidence documented in most of the articles reviewed. This discrepancy, however, may be attributed to missing data in several articles we studied [5,16], respectively.

Limitations to the study

Although all the articles we reviewed documented relevant risk factors, all the studies are observational. Again, the reviewed articles identified some risk factors, but most studies focused primarily on treatment modalities and diagnostic protocols. Risk factors were measured passively in these studies, so the impact and objective validity of identified risk factors are lacking. Moreover, although most articles concluded that females are far more affected than males, the articles failed to characterize other risk factors and commodities with gender specificity in mind. For example, if an article indicated that 60% of the population studied had DM, it would be more scientifically informative to predict how many are males and females, respectively.

Similarly, the population studied in most of the articles is Caucasians. There is no robust information on other races except in the study involving Maori and Chinese patients and the survey conducted by Omer et al. [16], where there is some emphasis on race classification. Thus, without similar studies involving significant other races, the evidence that Caucasians are more affected than others may be biased. In addition, data clarifying the medication patients were using at diagnosis is essential in determining if medications are the source of risk or complications. For example, there is no timeline for using VKA, iron, calcium phosphate binders, and vitamin D [5] when calciphylaxis was made. Again, some numeric values on our table were calculated by rounding off some numbers to the nearest decimal point and or converting percentages to numerical values. This adjustment may negatively affect our overall data accuracy. We also noted that most studies are observational with a small sample size, which may significantly affect the power of the studies and clinical relevance. Finally, as seen in some tables above, some data are unreported. Thus, the missing data might affect the accuracy of the risk frequency.

Relevance of the study

We identified and reviewed research conducted in six countries: the USA, France, Thailand, China, Australia, and New Zealand. We are particularly interested in the geographic spread of the studies, given that calciphylaxis is still an incompletely understood rare disease. Also, we need a better understanding of similarities and differences in risk factors across countries. Moreover, with its pooled sample size, this systematic review is more likely than individual studies to represent the risk profile accurately. Though challenging, we still believe in the possibility of multicentered experimental research involving a large sample size, as this is essential to characterize the risk factors and profile accurately. Such studies will optimize clinical suspicion and vigilance and encourage early diagnosis and prompt treatment, which may result in improved morbidity, mortality, and overall prognosis.

## Conclusions

Calciphylaxis has high mortality and morbidity potential coupled with poor treatment outcomes, even with a multimodal and multidisciplinary management protocol. Prevention is considered vital to achieving better treatment success. However, preventing the development of calciphylaxis significantly rests on accurately characterizing risk factors and risk profiles. Unfortunately, a comprehensive risk assessment and risk profiling are lacking. Besides, most of the studies in our review are observational and have a small sample size. Thus, we recommend a multicenter, large-scale experimental study on risk factors associated with the development of calciphylaxis in patients with chronic kidney diseases.
